# How to Apply the Sequential Correction Technique to Treatment of Congenital Cervicothoracic Scoliosis: A Technical Note and Case Series

**DOI:** 10.1111/os.70052

**Published:** 2025-05-19

**Authors:** Saihu Mao, Kai Sun, Song Li, Jie Zhou, Hongda Bao, Benlong Shi, Xu Sun, Zhen Liu, Yong Qiu, Zezhang Zhu

**Affiliations:** ^1^ Division of Spine Surgery, Department of Orthopedic Surgery, Nanjing Drum Tower Hospital, Affiliated Hospital of Medical School Nanjing University Nanjing China; ^2^ Division of Spine Surgery, Department of Orthopedic Surgery, Nanjing Drum Tower Hospital Affiliated Hospital of Nanjing Medical University Nanjing China

**Keywords:** congenital cervicothoracic scoliosis, hemivertebra resection, multi‐rod constructs, sequential correction technique, technical note

## Abstract

**Objective:**

Hemivertebrae in the cervicothoracic junction in the pediatric population are treated conventionally with a two‐rod instrumentation pattern. However, the increase in complexity, severity, and immaturity of osseous malformation in the cervicothoracic spine presents additional challenges in construct planning. This study aims to introduce an integrated instrumentation strategy named the sequential correction technique in the treatment of congenital cervicothoracic scoliosis caused by hemivertebra (CTS‐HV) and evaluate its feasibility and treatment effects.

**Methods:**

We retrospectively analyzed a consecutive series of patients with CTS‐HV who underwent posterior‐only HV resection with sequential correction technique from March 2018 to November 2023. This technique employed multiple rods, each being designated for a specific task, to sequentially perform surgical maneuvers involving osteotomy closure, torticollis correction, and implant integration. Individualized adjustments on instrumentation configuration involving rod number, rod type (whole, segmental, or satellite), cervical anchor choice, and connector placement could be made according to the severity of CTS and cervical pedicle dysplasia. Radiographic deformity parameters of the head–neck–shoulder complex were measured preoperatively, postoperatively, and at the latest follow‐up. One‐way repeated measures analysis of variance and Bonferroni correction were used to compare data at different time points. Additionally, any complications that occurred intraoperatively and during follow‐up would be recorded.

**Results:**

Twenty‐two pediatric and adolescent patients were recruited with a mean age of 8.3 ± 3.7 years. The ratio for the location of the resected CTS‐HVs were C6 (4.6%), C7 (13.6%), T1 (31.8%), T2 (9.1%), T3 (27.6%), and T4 (13.6%). All patients were instrumented with screw‐hook hybrid constructs, of which 3‐rod and 4‐rod constructs accounted for 81.8% and 18.2%, respectively. The cervicothoracic scoliosis, T1 tilt, neck tilt, clavicular angle, head tilt, and head shift were all significantly corrected from 53.1° ± 11.4°, 25.3° ± 10.1°, 19.6° ± 9.3°, 4.5° ± 3.1°, 10.7° ± 8.3°, and 21.8 ± 18.0 mm preoperatively to 20.8° ± 7.6°, 14.4° ± 7.2°, 7.3° ± 6.5°, 2.3° ± 2.6°, 4.4° ± 2.5°, and 9.8 ± 8.8 mm postoperatively (all *p* < 0.05). No significant correction loss was observed at the final follow‐up (all *p* > 0.05). The incidences of intraoperative dural tear and iatrogenic Horner's syndrome were both 4.6%. Transitory bilateral nerve root paralysis causing upper limb dysfunction occurred in 1 patient. Additionally, 3 patients suffered severe distal curve progression with trunk tilt and were surgically revised with instrumentation extending to the stable zone. No implant‐related complications were observed.

**Conclusions:**

This modified sequential correction technique possesses the merits of easy rod installation, satisfying torticollis correction, good symmetry and verticality of the entire instrumentation, and high fixation rigidity with multi‐rod constructs across the cervicothoracic junction. Thus, it is endowed with great application values in the treatment of CTS.

## Graphical abstract

Sequential Correction Technique for Congenital Cervicothoracic Scoliosis. A novel multi‐rod construct technique offering effective deformity correction, enhanced symmetry, and robust fixation stability.

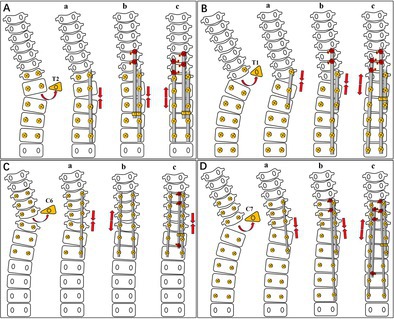

## 1 Introduction

Congenital cervicothoracic scoliosis (CTS) caused by hemivertebra (HV) located in the cervicothoracic junctional area is rare but frequently results in significant appearance deformities in early childhood, including neck tilt, head deviation, shoulder imbalance, and facial asymmetry [1, 2, 3]. Early HV resection with conventional two‐rod constructs was reported to be effective for correcting the torticollis and balancing the shoulders [4, 5, 6, 7, 8, 9]. However, additional challenges in construct planning may be encountered when dealing with early‐onset immature pediatric patients with severe and complicated cervicothoracic deformities.

The symmetry and verticality of instrumentation is the premise of pursuing long‐lasting satisfactory curve correction. Thus, placing fixation anchors in the lower cervical spine is highly necessary in the treatment of CTS patients, particularly for torticollis correction. However, due to the technical complexity, as well as abnormal anatomy and immature osseous structure, cervical pedicle screws (CPS) may not always be implantable due to the high risk of screw malposition, and may need to be replaced with lamina hooks. The anatomical malalignment between the anchor points of the cervical lamina hooks and the upper thoracic pedicle screws, however, can make rod contouring and installation challenging.

In cases of successful CPS placement with the aid of O‐arm navigation, a severe angular CTS caused by multi‐level osseous defects is another scenario in which the anchor points for pedicle screws above and below the HV often show significant malalignment, making it not very amenable to traditional 2‐rod corrections. Additionally, the biomechanical studies indicated that the cervicothoracic spine is a high‐stress concentration area [10]. HV resection can further compound the inherent instability of this junctional area [11]. As reported by Wang, 2 out of 25 CTS patients experienced rod fracture following cervicothoracic spinal surgery, with an incidence rate as high as 8.0% [6]. This underscores the critical need for robust strength and stability of the internal fixation in the cervicothoracic spine.

For the treatment of refractory spinal deformities, the sequential correction technique proposed by Zhu simplifies the rod installation through step‐by‐step separated operative maneuvers with multi‐rod constructs [12]. Its superiority in correcting idiopathic, de novo, and congenital structural spinal deformities has been well reported [12, 13, 14]. This seems intuitive for the treatment of CTS as such pediatric patients may be alternatively managed by this novel technique. Since 2018, the sequential correction technique has been employed to treat CTS patients in our scoliosis center. This study aims to outline the modified surgical procedures in detail, assess the feasibility and short‐term surgical outcomes, and clarify the advantages of this technique in the treatment of CTS.

## 2 Materials and Methods

### 2.1 Patients and Baseline Evaluation

After approval from the institutional review board (No. of 2021‐398‐01), a series of pediatric and adolescent patients undergoing corrective surgery for CTS at our center were retrospectively reviewed. Inclusion criteria were: (1) cervicothoracic HV located at C6‐T4; (2) significant cervicothoracic scoliosis ≥ 25° with torticollis, facial asymmetry, or shoulder imbalance; (3) underwent sequential correction technique with multi‐rod constructs; (4) follow‐up more than 1 year. Exclusion criteria included the following: (1) history of spine surgery; (2) suffering from neurological deficits; (3) Sprengel deformity; (4) lower instrumented vertebra (LIV) distal to T9; (5) hybrid growing rod technique. Eventually, a general screen of our scoliosis database identified 22 CTS patients who fit the inclusion and exclusion criteria between March 2018 and November 2023. The demographic data of patients including age, sex, additional skeletal malformation, level of excised HV, instrumentation type, range of fusion, operative time, blood loss, and complications were recorded.

Preoperatively, all patients underwent comprehensive assessments, including standing full‐spine X‐rays, whole‐spine CT scans with three‐dimensional reconstruction, and magnetic resonance imaging (MRI). If necessary and feasible, preoperative cervicothoracic 3D‐printed models would be created to help improve the understanding of the structural malformation and facilitate the design of the surgical strategy. Since 2018, the modified sequential correction technique has been employed to treat CTS patients with the following indications: (1) severe CTS with significant misalignment between the lower cervical and upper thoracic spine, which makes it not very amenable to traditional 2‐rod corrections despite successful CPS placement with the aid of O‐arm navigation; (2) high risks of implantation of CPS because of the abnormal anatomy or significant immaturity of the cervical pedicles, and meanwhile the lack of O‐arm navigation support. Usually, the cervical lamina hook (CLH) rather than lateral mass screw would be preferentially used as a substitute for CPS, increasing the torticollis correction as well as the symmetry and verticality of the entire instrumentation. In this situation, meeting the high demands for accurate contouring and installation of two long rods using screw‐hook hybrid constructs was quite a challenge. The modified sequential correction technique with multi‐rod constructs was thus introduced to tackle these problems.

### 2.2 The Sequential Correction Technique

After inducing general anesthesia, the patients were positioned prone on a radiolucent Jackson table. If the CPS placement was planned, the patient would be applied with the Mayfield skull clamp and the cranial reference frame to facilitate O‐arm navigation. A posterior midline approach to the cervicothoracic region was utilized, systematically dissecting the skin, fascia, and paraspinal muscles to expose the targeted surgical area. Pedicle screws were inserted sequentially above and below the target hemivertebra using a conventional free‐hand technique or under the guidance of O‐arm navigation. In case of infeasibility or failure of CPS insertion for extremely dysplastic or malformed pedicles, CLH was utilized as a substitute anchor. Afterward, somatosensory evoked potentials (SSEPs) and transcranial electrical motor evoked potentials (TCeMEPs) would be performed to confirm the integrity of neurological function. If uneventful, apical hemivertebra resection would be performed as planned. After completion of the HV excision, the individualized sequential correction technique with multi‐rod constructs would be employed according to the type and distribution of spinal anchors and deformity characteristics.

**Scenario 1:** Infeasibility or failure of CPS placement due to pedicle malformation or technical limitations, and CLH were used as substitutes. A low‐profile instrumentation system based on the 4.5 mm or 5.5 mm rod diameter was usually utilized.

**FIGURE 1:**
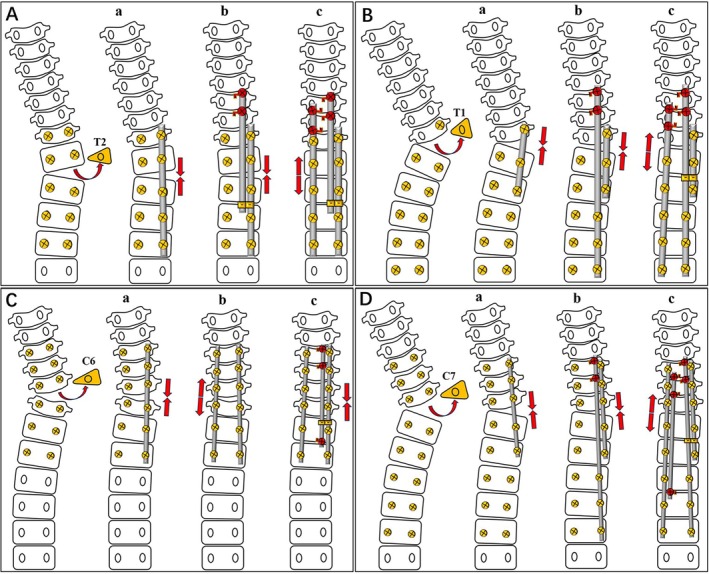
Schematic diagrams of the four different patterns of the sequential correction technique.

**FIGURE 2:**
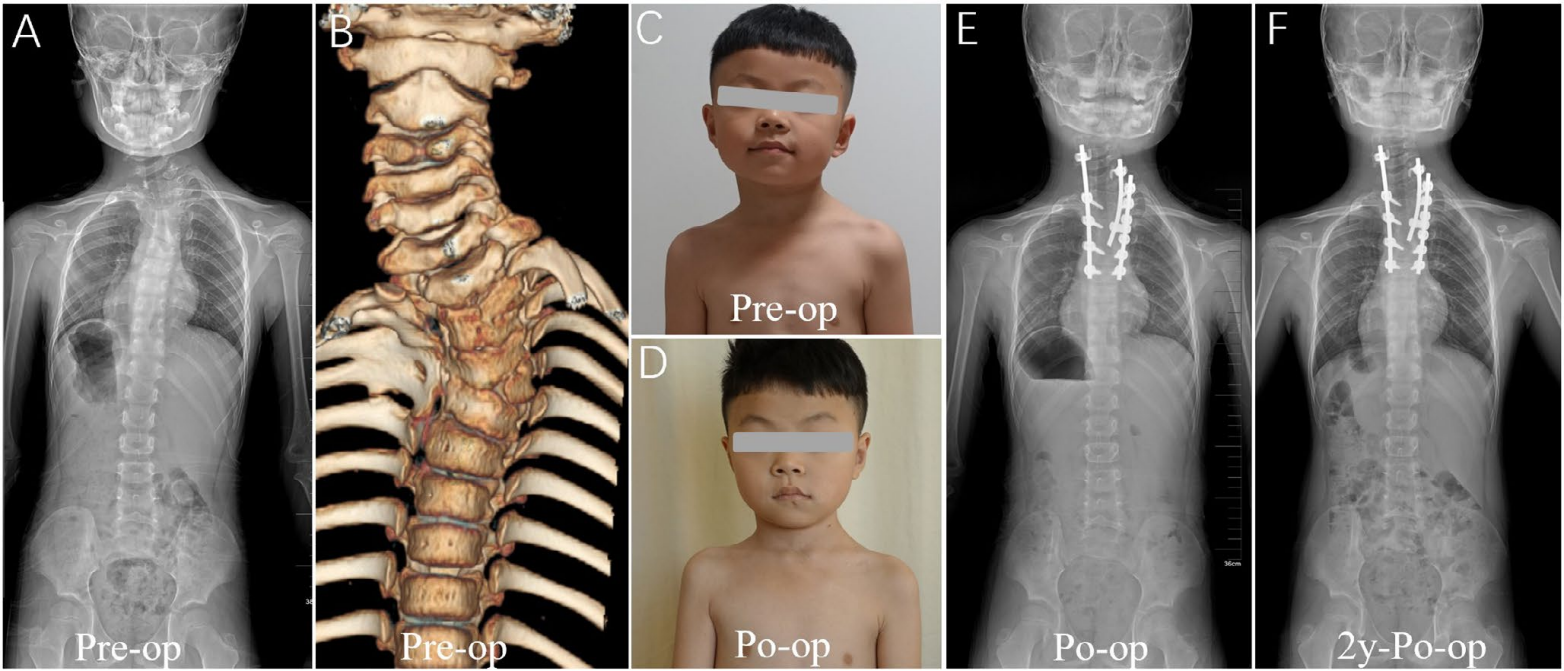
Pattern A. (A–C): The case of No. 1 is a 7‐year‐old boy with HV located at T4. (D,E): At the age of 7, he underwent posterior T4 hemivertebra resection and C5‐T7 internal fixation at our hospital. Initially, a rod was installed on the convex side (T1‐T7) to close the osteotomy site and achieve preliminary correction. Subsequently, CLH was utilized to extend the fixation upward, achieving further correction of torticollis. (F): At the two‐year follow‐up, postoperative X‐rays showed that the corrective outcome was well maintained.

Pattern A. The malalignment of spinal anchors was moderate grade, and the fusion segments were relatively short (Figures 1A and 2): a. Install a segmental rod on the convex side to close the osteotomy gap. The proximal end of this rod was usually 1–2 levels cranial to HV, and the distal end stopped at the stable zone. b. Install another separate rod medially on the convex side, which extends proximally to the upper instrumented vertebra (UIV) anchored by CLH. A rod‐to‐rod connector was used to achieve a connection between the distal terminal and the first rod. Proximal compression maneuvers were performed to increase the torticollis correction. c. Install a long rod on the concave side with the proximal end being anchored by CLH, and distraction was carried out to further increase the correction of CTS and torticollis.

**FIGURE 3:**
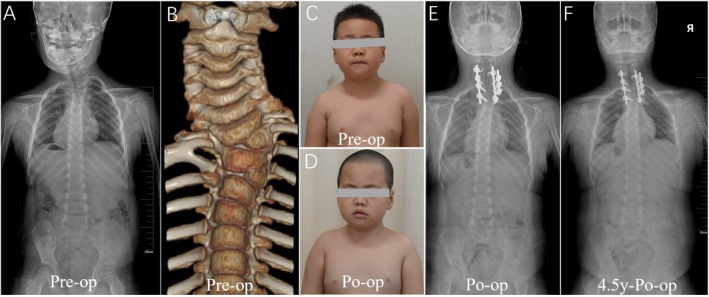
Pattern B. (A–C): The case of No. 11 is a 6‐year‐old boy with HV located at T1 and T3. (D,E): At the age of 6, he underwent posterior T1 HV resection and C6‐T5 internal fixation in our hospital. The procedure began with the placement of a short rod at C7‐T4 to close the osteotomy site, followed by the sequential installation of two additional rods on the convex and concave sides to achieve further deformity correction. (F): At the 4.5‐year follow‐up, postoperative X‐rays confirmed that the corrective outcome was well maintained.

Pattern B. The malalignment of spinal anchors was high‐grade and the fusion segments were relatively long (Figures 1B and 3). a. Install a short rod on the convex side for osteotomy site closure, usually presenting as 1 or 2 levels centering around HV. b. Install another convex long rod medial to the short rod. The proximal and distal rod ends were anchored by CLH and pedicle screws, respectively. Both ends were compressed and shortened for correction of CTS and torticollis. c. Install a long rod on the concave side. Concave distraction maneuvers being performed on proximal cranially oriented hooks and distal pedicle screws were beneficial for further correction of CTS and torticollis.

**Scenario 2:** Total or partial success of CPS placement with the aid of O‐arm navigation. A cervical instrumentation system based on the 3.2 mm or 3.5‐mm rod diameter or cervical‐thoracic hybrid constructs using a connector or a transitional rod was the two main options of instrumentation.

**FIGURE 4:**
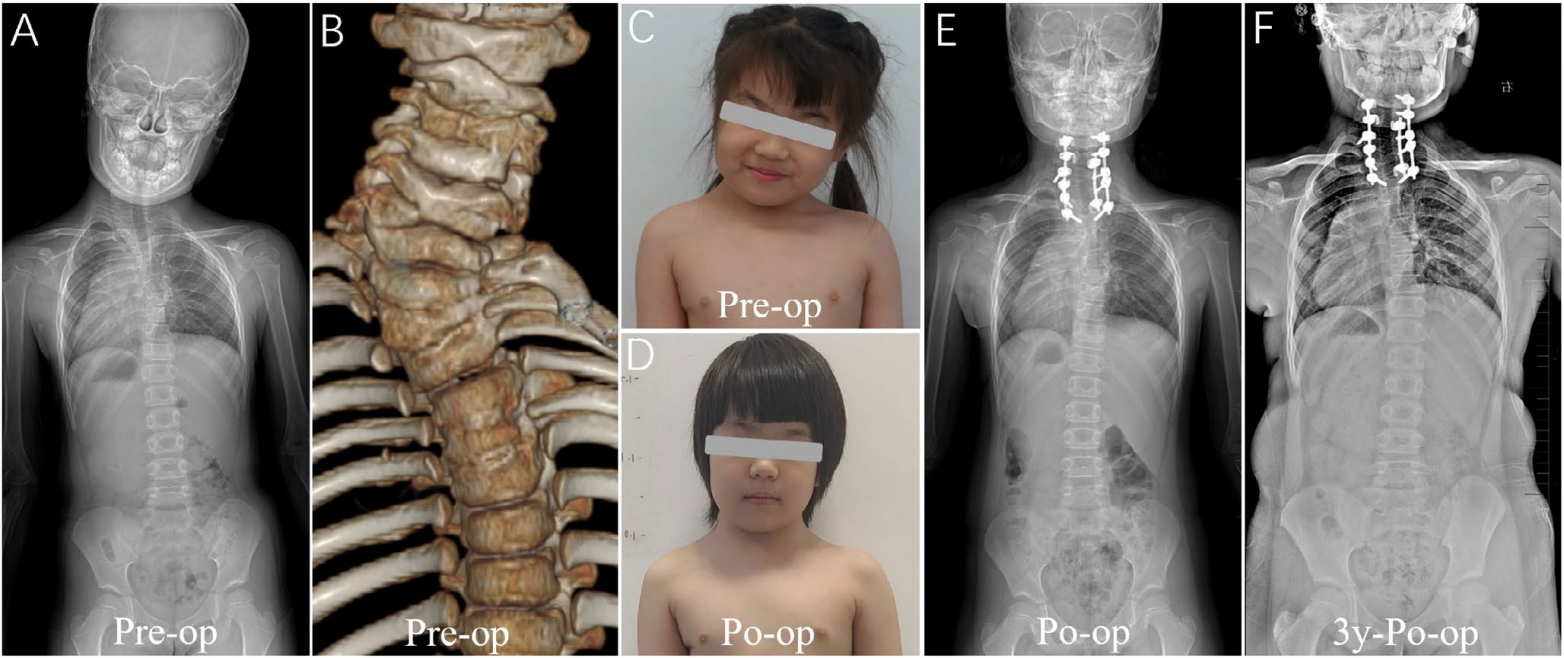
Pattern C. (A–C): The case of No. 20 involves a 6‐year‐old girl diagnosed with C7 HV and severe Klippel‐Feil Syndrome. (D,E): At the age of 6, she underwent posterior C7 HV resection and C3‐T3 internal fixation. After the sequential implantation of three rods, the local deformity was well corrected. (F): The corrective outcome was well maintained at the three‐year follow‐up.

Pattern C. The malalignment of spinal anchors was moderate‐grade and the fusion segments were relatively short (Figures 1C and 4). a, b. Install two long rods sequentially on both convex and concave sides along with convex compression/concave distraction maneuvers for osteotomy closure and CTS correction. c. Install an additional satellite rod medially on either the convex or concave side to further strengthen the rigidity of fixation and correction of torticollis. This satellite rod was anchored with lamina hooks at both ends.

**FIGURE 5:**
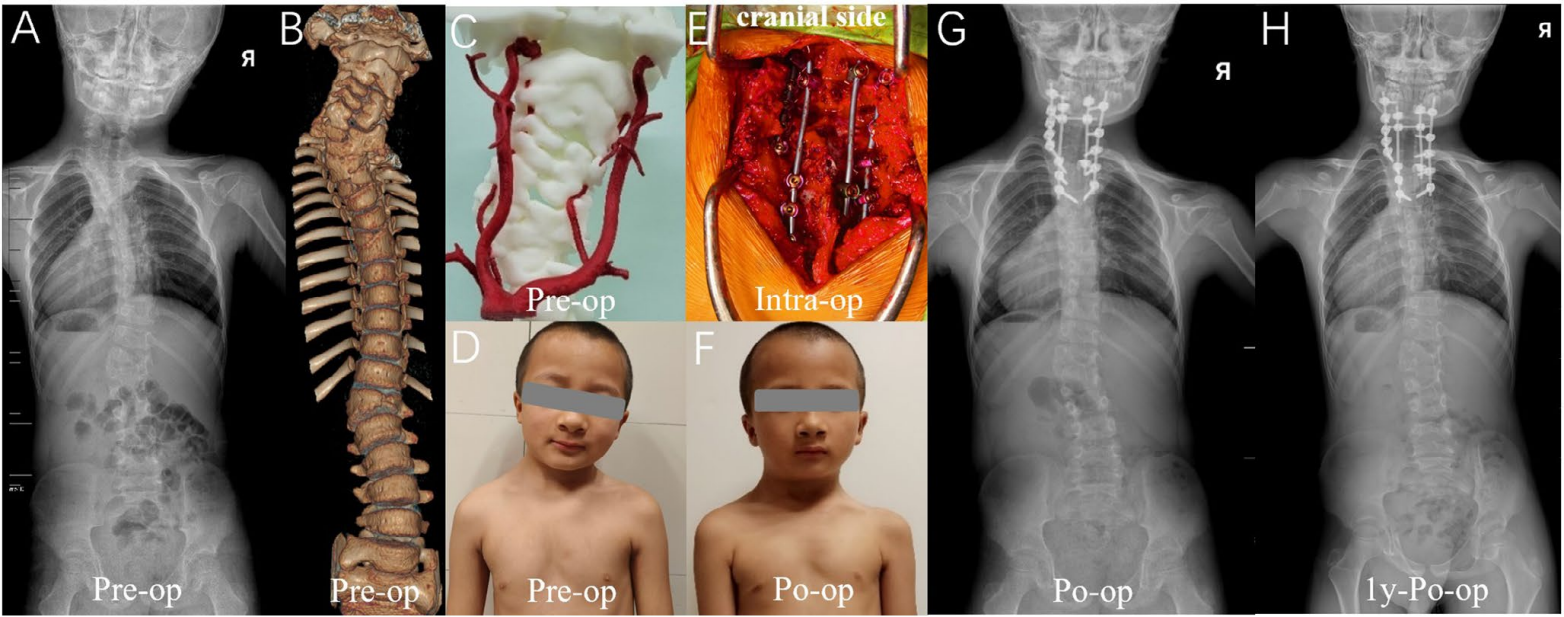
Pattern D. (A–D): The case of NO. 21 is an 8‐year‐old boy with significant torticollis and head tilt. The HVs are located at the C7 and T1 levels, and he also presented with multi‐level segmentation failure and Klippel‐Feil Syndrome. (E): He underwent posterior C7 HV resection and C4‐T5 internal fixation with the application of the sequential correction technique. (F–H): Significant improvement in the patient's appearance can be observed postoperatively and was well maintained during follow‐up.

Pattern D. The malalignment of spinal anchors was high‐grade, and the fusion segments were relatively long (Figures 1D and 5). a. Install a segmental rod centering around the HV on the convex side to close the osteotomy gap. b. Install another segmental rod medial to the first rod on the convex side. The rod was anchored by the residual distal convex pedicle screws and the proximal CLH. c. Install a third rod on the concave side with concave distraction, and install a fourth concave satellite rod medially to add strength to the overall constructs.

Despite these aforementioned outlined scenarios, individualized adjustments on instrumentation configuration involving rod number, rod type (whole, segmental, or satellite), cervical anchor choice, and connector placement could be made according to the actual intraoperative situation. By coordinating and integrating these implants, torticollis correction and alignment control could be achieved step by step. Autogenous and/or allogeneic bone grafting was then performed, followed by drainage placement and incision closure. Neurophysiological monitoring involving SSEPs and TCeMEPs would be performed again in case of unanticipated neurological deficits. Rigid neck collar immobilization was routinely prescribed for 3 months after the surgery.

### 2.3 Radiographic Measurements

Given the presence of mandibular occlusion, the Cobb angle in the cervicothoracic area was measured on 3D‐CT reconstruction. (1) Segmental scoliosis: the angle between the superior endplate of the upper‐end vertebra and the inferior endplate of the lower‐end vertebra in the major cervicothoracic curve [15]. (2) Osteotomy correction rate: The Cobb angle at the osteotomy site, measured preoperatively (Cobb_1_) and postoperatively (Cobb_2_), was defined as the angle between the lines being parallel to the superior endplate of the proximal vertebra neighboring the hemivertebra and the inferior endplate of the neighboring vertebra below. The osteotomy correction rate was calculated as (Cobb_1_ – Cobb_2_) / Cobb_1_.

**FIGURE 6:**
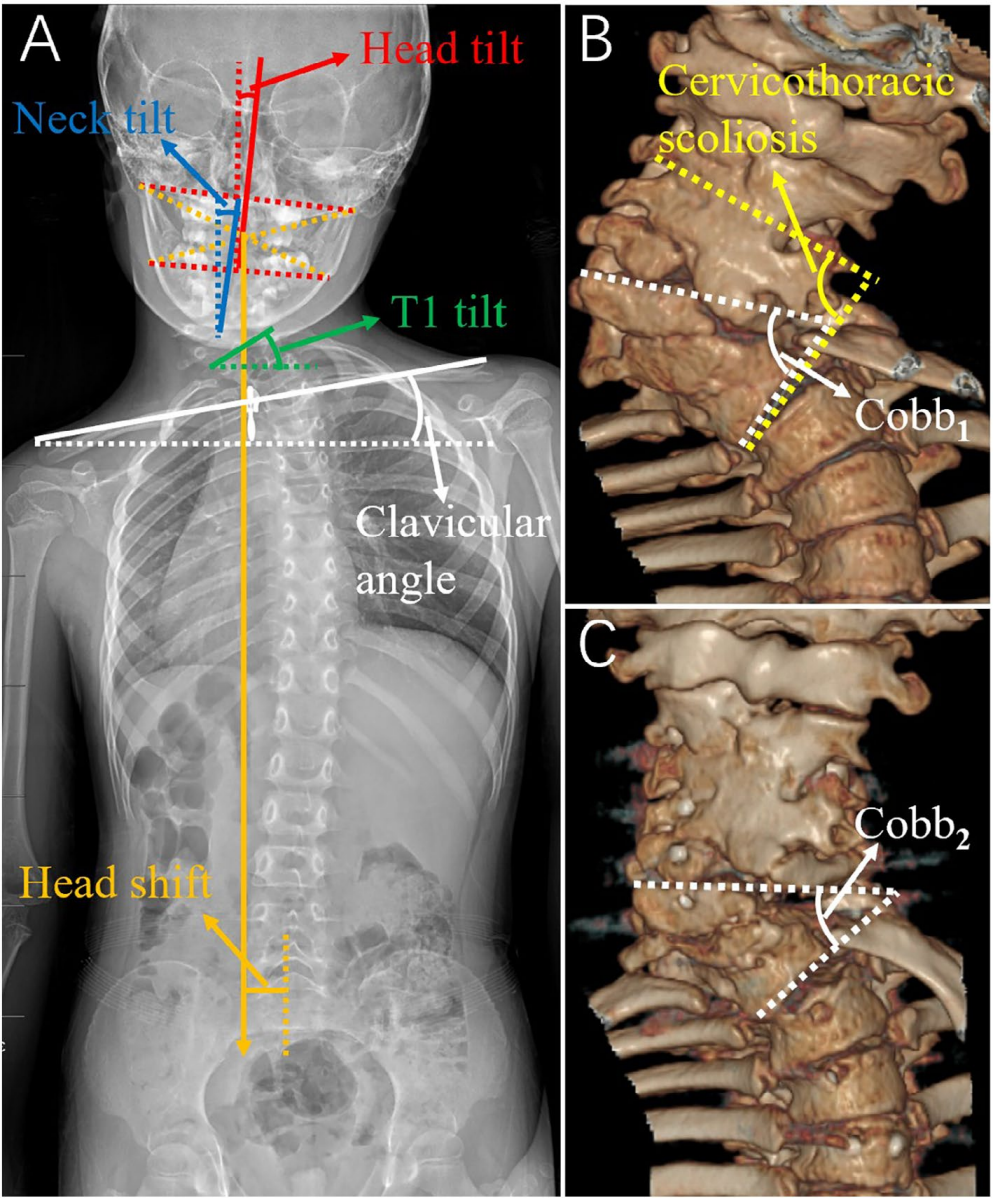
Schematic illustration of radiographic measurements of different deformity parameters.

The following parameters were measured on standing whole‐spine X‐rays. (1) T1 tilt: the angle between the T1 vertebra's upper endplate and the horizontal line [15]. (2) Neck tilt: the angle between the C7 vertebra center to the C2 odontoid process line and the vertical line [15]. (3) Clavicular angle: the angle between the horizontal line and the tangential line connecting the highest two points of each clavicle [15]. (4) Head tilt: the angle between the long axis of the head and the vertical line. Two lines connecting the bilateral temporomandibular joints and the mandibular angle were drawn separately, and the longitudinal axis of the head was defined as the line connecting the midpoints of the above two lines [15]. (5) Head shift: the horizontal distance between the head's central point and the central sacral vertical line. The center point of the head was defined as the intersection of the diagonals of the temporomandibular joint and the mandibular angle [15] (Figure 6).

The above parameters were independently measured by two experienced spine surgeons, and the average value was taken. The corrective effect of segmental scoliosis, T1 tilt, neck tilt, clavicular angle, head tilt, and head shift was then evaluated by the two surgeons.

### 2.4 Statistical Analysis

Statistical analyses were performed using SPSS version 26.0 (IBM, UAS). The Kappa value was used to assess the consistency of correction effect evaluations between the two surgeons. A value of 0.75–1 indicates excellent consistency, 0.40–0.75 indicates moderate consistency, and 0–0.40 indicates poor consistency. One‐way repeated measures analysis of variance (ANOVA) was used to compare data at different time points. Post hoc analysis was performed using the Bonferroni correction for pairwise comparisons. *P* value < 0.05 was considered statistically significant.

## 3 Results

### 3.1 General Data

A total of 11 males and 11 females were recruited, and the age averaged 8.3 ± 3.7 years. The location of excised cervicothoracic HVs was C6 in 1 case, C7 in 3 cases, T1 in 7 cases, T2 in 2 cases, T3 in 6 cases, and T4 in 3 cases. The number of fused segments averaged 9.1 ± 2.0. The mean follow‐up time was 29.9 ± 19.1 months. The operating time and blood loss averaged 275 ± 56 min and 673 ± 340 mL, respectively.

### 3.2 Instrumentation

Fifteen patients were under Scenario 1, fully utilizing the thoracic instrumentation system (4.5 mm or 5.5 mm). The fixation style was Pattern A in 9 cases and Pattern B in 6 cases. Seven patients fell under Scenario 2, utilizing a complete cervical instrumentation system (3.2 mm or 3.5 mm). Among them, the fixation style was Pattern C for 5 cases and Pattern D for 2 cases. All patients underwent multi‐rod and screw‐hook hybrid constructs, with 4‐rod constructs applied in 4 cases (one from each pattern) and 3‐rod constructs in the remaining 18 cases (Table 1).

**TABLE 1:** Demographic and operative data of the recruited patients.

Case	Sex and age (y)	Resected HV	Other concomitant malformation	Type of instrumentation	Instrumentation	Operating time (min)	Blood loss (mL)	Complications
1*	M, 7	T4 (R)	C3 BV, T1‐T4 SF	A	R: T1‐T7 (TPS), 4.5 mm R: C7‐T4 (CLH + RTR), 4.5 mm L: C5‐T7 (CLH + TPS) 4.5 mm	270	400	Horner syndrome
2	F, 12	T4 (L)	C2‐C3 SF, C7 HV(R), C7‐T5 SF	A	L: T1‐T7 (TPS), 5.5 mm L: C6‐T4 (CLH + RTR), 5.5 mm R: C6‐T7 (CLH + TPS) 5.5 mm	245	800	—
3	M, 5	T1 (R)	C7 BV, T2 BV, T5 HV (R)	A	R: C7‐T7 (CTP + TPS), 4.5 mm R: C6‐T2 (CLH + RTR), 4.5 mm L: C6‐T7 (CLH + TPS) 4.5 mm	275	1100	—
4	F, 7	T3 (R)	T2‐T4 SF	A	R: T1‐T5 (TPS), 4.5 mm R: C6‐T3 (CLH + RTR), 4.5 mm L: C6‐T5 (CLH + TPS) 4.5 mm	240	500	—
5	M, 4	T3 (L)	C7‐T5 SF	A	L: T1‐T6 (TPS), 4.5 mm L: C6‐T3 (CLH + RTR), 4.5 mm R: C6‐T6 (CLH + TPS), 4.5 mm	205	400	Dural tear
6	M, 13	T1 (L)	C7‐T4 SF, T4 BV	A	L: C7‐T5 (CPS + TPS), 4.5 mm L: C6‐T3 (CLH + RTR), 4.5 mm R: C7‐T5 (CPS + TPS), 4.5 mm	240	800	—
7	M, 6	T4 (L)	C7‐T5 SF	A	L: T1‐T6 (TPS), 4.5 mm L: C6‐T7 (CLH + RTR), 4.5 mm R: C6‐T7 (CLH + TPS) 4.5 mm	215	300	—
8	F, 11	T2 (R)	C2‐T6 SF, C3 BV	A	R: T1‐T6 (TPS), 5.5 mm R: C4‐T4 (CLH + RTR), 5.5 mm L: C4‐T6 (CLH + TPS) 5.5 mm	295	600	Revision surgery for distal curve progression
9	F, 15	T3 (L)	C6 HV (R)	A	L: T1‐T8 (TPS), 5.5 mm L: C5‐T6 (CLH + RTR), 5.5 mm R: C5‐T4 (CLH + TPS), 5.5 mm R: T1‐T8 (TPS) 5.5 mm	300	800	—
10	F, 6	T3 (R)	C6 HV (R), C6‐T4 SF	B	R: T2‐T4 (TPS), 4.5 mm R: C6‐T7 (CLH + TPS), 4.5 mm L: C6‐T7 (CLH + TPS) 4.5 mm	245	600	—
11*	M, 6	T1 (R)	T3 HV (R)	B	R: C7‐T4 (CPS + TPS), 4.5 mm R:C6‐T5 (CLH + TPS), 4.5 mm L: C6‐T5 (CLH + TPS) 4.5 mm	160	200	—
12	M, 4	T3 (L)	C5‐T4 SF	B	L: T1‐T4 (TPS), 4.5 mm L: C6‐T7 (CLH + TPS), 4.5 mm R: C6‐T7 (CLH + TPS) 4.5 mm	255	800	—
13	F, 5	T2 (L)	C2‐C3 SF, C5‐C7 SF, T3‐T6 SF, T8‐T10 SF	B	L: T1‐T4 (TPS), 4.5 mm L: C6‐T5 (CLH + TPS), 4.5 mm R: C6‐T5 (CLH + TPS) 4.5 mm	175	700	Revision surgery for distal curve progression
14	F, 12	T3 (R)	C1‐T5 SF	B	R: T2‐T4 (TPS), 5.5 mm R: C6‐T8 (CLH + TPS), 5.5 mm L: C4‐T8 (CLH + TPS) 5.5 mm	350	1000	Revision surgery for distal curve progression
15	M, 13	T1 (R)	C4‐T6 SF	B	R: T1‐T4 (TPS), 5.5 mm R: C7‐T11 (CPS + TPS), 5.5 mm L: C5‐T4(CLH + TPS), 5.5 mm L:C7‐T11 (CPS + TPS) 5.5 mm	360	1600	—
16	M, 15	C6 (L)	L1‐L4 SF	C	L: C5‐T2 (CPS + TPS), 3.5 mm L: C5‐T2 (CLH + TLH), 3.5 mm R:C5‐T2 (CPS + TPS) 3.5 mm	320	800	—
17	F, 9	T1 (R)	C2‐C3 SF, C4‐C5 SF, C6‐T6 SF, T9‐T10 SF, L1 HV (R)	C	R: C3‐T5 (CPS + TPS), 4.75 mm R: C3‐T5 (CLH + TLH), 3.2 mm L: C3‐T5 (CPS + TPS), 4.75 mm L: C3‐T5 (CLH + TLH) 3.2 mm	290	1000	—
18	F, 6	T1 (R)	C4‐T3 SF, T4 HV (R)	C	R: C4‐T7 (CPS + TPS), 3.2 mm L: C4‐T7 (CPS + TPS), 3.2 mm L: C4‐T6 (CLH + TLH) 3.2 mm	325	300	—
19	M,3	T1 (L)	C6‐T3 SF, C3 BV, C6BV, T3BV	C	L: C4‐T4(CPS + TPS), 3.2 mm R:C4‐T4 (CPS + TPS), 3.2 mm R: C4‐T4 (CLH + TLH) 3.2 mm	310	300	—
20*	F, 6	C7 (L)	C2‐C4 SF, C5‐C7 SF, T1‐T3 SF, T4‐T6 SF	C	L: C3‐T3 (CPS + TPS), 3.2 mm R: C3‐T3 (CPS + TPS), 3.2 mm R: C4‐T3(CLH + TLH) 3.2 mm	330	400	—
21*	M, 7	C7 (L)	C2‐T3 SF, C3‐C6 BV T6‐T7 SF, L1 HV (L)	D	L: C4‐T2 (CPS + TPS), 3.2 mm L: C5‐T5 (CLH + TPS), 3.2 mm R: C4‐T3 (CLH + TLH), 3.2 mm R:C4‐T5(CPS + TPS) 3.2 mm	320	1000	—
22	F, 10	C7 (L)	C2‐T1 SF	D	L: C2‐T2 (CPS + TPS), 3.2 mm L: C6‐T5 (CLH + TPS), 3.2 mm R: C2‐T5 (CPS + TPS), 3.2 mm	335	400	Bilateral nerve root paralysis

Abbreviations: *, demo case; BV, butterfly vertebra; CLH, cervical lamina hook; CPS, cervical pedicle screw; F, female; HV, hemivertebra; L, left; M, men; R, right; RTR, rod‐to‐rod connector; SF, segmentation failure; TLH, thoracic lamina hook; TPS, thoracic pedicle screw.

### 3.3 Radiographic Measurements

The deformity parameters, including cervicothoracic scoliosis, T1 tilt, neck tilt, clavicular angle, head tilt, and head shift, were significantly corrected from 53.1° ± 11.4°, 25.3° ± 10.1°, 19.6° ± 9.3°, 4.5° ± 3.1°, 10.7° ± 8.3°, and 21.8 ± 18.0 mm preoperatively to 20.8° ± 7.6°, 14.4° ± 7.2°, 7.3° ± 6.5°, 2.3° ± 2.6°, 4.4° ± 2.5°, and 9.8 ± 8.8 mm postoperatively (all *p* < 0.05). The Cobb angle at the osteotomy site was reduced from 42.2° to 22.8°, achieving an osteotomy correction rate of 46.0%. At the last follow‐up, no significant differences were observed compared to the postoperative measurements; the deformity parameters remained stable at 21.7° ± 7.5°, 14.8° ± 7.2°, 8.4° ± 7.2°, 2.2° ± 2.7°, 4.5° ± 3.2°, and 12.4 ± 10.3 mm, respectively (all *p* > 0.05) (Table 2).

**TABLE 2:** Comparison of radiographic deformity parameters before and after surgery.

	Pre‐operative	3 months post‐operative	Last follow‐up	*F*‐value, *p*‐value
Segmental scoliosis (°)	53.1 ± 11.4	20.8 ± 7.6^a^	21.7 ± 7.5^a^	*F* = 249.970, *p* < 0.001
T1 tilt (°)	25.3 ± 10.1	14.4 ± 7.2^a^	14.8 ± 7.2^a^	*F* = 60.739, *p* < 0.001
Neck tilt (°)	19.6 ± 9.3	7.3 ± 6.5^a^	8.4 ± 7.2^a^	*F* = 76.092, *p* < 0.001
Clavicular angle (°)	4.5 ± 3.1	2.3 ± 2.6^a^	2.2 ± 2.7^a^	*F* = 22.804, *p* < 0.001
Head tilt (°)	10.7 ± 8.3	4.4 ± 2.5^a^	4.5 ± 3.2^a^	*F* = 15.020, *p* = 0.001
Head shift (mm)	21.8 ± 18.0	9.8 ± 8.8^a^	12.4 ± 10.3^a^	*F* = 8786, *p* = 0.002

^a^ Compare to the pre‐operative, *p* < 0.05.

### 3.4 Consistency Test

The consistency test showed excellent consistency between two observers in evaluating the cervicothoracic deformities correction effect based on radiographic parameters (segmental scoliosis, T1 tilt, neck tilt, clavicular angle, head tilt, and head shift), with all Kappa values > 0.75.

### 3.5 Complications

One patient experienced incidental dural tear and suffered no neurological sequelae after dural repair. Another patient developed Horner's syndrome due to intraoperative sympathetic nerve injury, presenting with unilateral miosis, ptosis, and anhidrosis of the right face. It resolved spontaneously 3 months after surgery. A third child patient suffered bilateral nerve root paralysis and reduced muscle strength in upper limbs, which improved significantly with rehabilitation. In addition, three patients with preoperative trunk tilt experienced severe distal curve progression postoperatively and underwent revision surgery to extend the LIV to the stable region. At the latest follow‐up, no implant‐related complications were observed.

## 4 Discussion

### 4.1 Sequential Correction Technique for Easy Rod Installation

When tackling CTS, the emerging challenge being recognized is that rod installation may be quite difficult with the conventional two‐rod instrumentation. Factors involving the malalignment of different spinal anchor types, angular CTS with multi‐level segmentation failure of neighboring vertebrae, rod bending inaccuracy, and the lack of a long‐tailed cervical pedicle screw/transitional rod/transitional connector may all hinder the smooth rod installation crossing the cervicothoracic junction.

Multi‐rod construct is an area of rapid proliferation in the treatment of refractory spinal deformities. In 2019, the concept of the sequential correction technique was developed by Zhu. This novel technique decomposes the complex process of rod installation into sequential steps, and each step is assigned only one task [12]. This simplified workflow is highly practical and has the potential to revolutionize the way of rod installation in the treatment of complex CTS. In the current study, we set up a flow of sequencing surgical procedures to ensure easier rod installation and denser rod configuration. Based on the feasibility of CPS implantation, we categorized the application scenarios of the modified sequential correction technique into two groups and further refined them into four patterns to tackle the varying degrees of difficulties we come across. Postoperatively, the mean correction rate of cervicothoracic scoliosis reached 60.8%, aligning with the range of 40%–80% reported in previous studies [5, 6, 7, 8, 9]. Additionally, T1 tilt, neck tilt, clavicular angle, head tilt, and head shift also showed satisfactory improvement.

The superiority of this technique lies in that it can stabilize the osteotomy region with a segmental rod in a quick fashion, reducing the bleeding of the epidural venous plexus, the difficulty of rod contouring and installation, the risk of cord injury as well as dispersing the stress of the implant/bone interface. Moreover, after initial correction in the apical region, proximal convex compression/concave distraction with a satellite rod and laminar hooks in the proximal cervical spine can further adjust and improve the torticollis correction when the apical osteotomy is insufficient because of technical limitations [9, 16]. It is also beneficial for achieving the symmetry and verticality of the entire instrumentation.

### 4.2 Advantages of Application of CLH in Sequential Correction Technique

CLH, rather than lateral mass screw, is selected as a vital component of the sequential correction technique. This is because the cervical lateral mass in the pediatric population is too small to achieve a reliable fixation. The CLH, however, possesses advantages of easy installation, reliable fixation strength, and lower risk of vertebral artery and nerve root injury, thus enabling it to be an ideal substitute for CPS. A key tip for the installation of CLH is that it should be cranially oriented on the concave side and caudally oriented on the convex side. The application of CLH can also guarantee the construction of a multi‐rod system even if the ratio of successful CPS placement is low. This is extremely important as the cervicothoracic junction is an area of concentrated stress, and HV resection can further exacerbate the instability in this region. Wang [6] reported on 25 CTS patients who underwent C7‐T1 three‐column osteotomy with traditional two‐rod instrumentation, demonstrating a rod fracture incidence of 8.0%. Similarly, research by Chakravarthy [17], Yang [18], and Deviren [19] identified incidences of screw pullout ranging from 2.2% to 8.9% and rod fracture between 0.7% and 9.1% after cervicothoracic fixation. To reduce the incidence of internal fixation failure, Hyun suggests that installing three rods following osteotomy can markedly decrease the risks of internal fixation failure and pseudoarthrosis [20]. Tatsumi also recommends using larger diameter thoracic screws and rods in clinical scenarios with expected high loads on the cervicothoracic spine [21]. In this study, all patients exhibited well stability of internal fixation, with no occurrence of screw/hook/rod pullout or fracture after surgery.

### 4.3 Current Dilemma and Recent Advancement

The primary therapeutic principle for the treatment of the congenital cervicothoracic hemivertebra is to correct the regional scoliosis and restore the head–neck–shoulder balance utilizing osteotomy, fixation, and fusion as short as possible. However, surgical manipulation is still a challenge since it demands early surgical intervention in the pediatric population with complicated and risky 3‐column spinal osteotomy(3CO) in the cervicothoracic junction. With respect to these considerations, it is highly likely that proper operative intervention could largely be delayed or ignored for a considerable number of such child patients worldwide. A promising hope for ways out of the current dilemma is to establish a general consensus providing answers to when and how such complicated surgery should be performed.

Recent advancements in studies concerning the natural evolution of curve morphology of CTS have indicated that severe trunk tilt with coronal imbalance (Type B coronal curve morphology) or caudal compensatory thoracic curve (Type C) could occur frequently with age if left untreated, especially for those with multi‐level osseous defects involving Klippel‐Feil Syndrome in particular [15, 22]. Under these circumstances, the apical cervicothoracic HV resection with short spinal fusion, which is the mainstream strategy in the current era [9], may not be likely to obtain a long‐lasting correction efficiency. As reported by Wang, 7 of 25 CTS patients experienced a distal curve progression of more than 10° after surgery, and 2 (8.0%) eventually required revision surgery [6]. Similarly, Huang and Burkhardt reported that 2/21 (9.5%) and 2/17 (11.7%) CTS patients, respectively, underwent revision surgery for progressive distal curves [5, 7]. In this study, three patients underwent revision surgery, and all three had a preoperative coronal pattern classified as Type B. Thus, it is gradually recognized that surgical intervention before the onset and rapid advance of Type B or C CTS is essential for the sake of effective deformity correction with short fixation and avoidance of a future revision or staged surgery in high probability.

Hemivertebra resection with circumferential release is the premise of satisfying correction of the rigid structural CTS, which is technically demanding. Due to the complex anatomy of the cervicothoracic junction with abundant adjacent blood vessels and nerves, as well as slender and often dysplastic cervical pedicles, procedures such as osteotomy and screw placement carry a high risk of vascular and neurological injury. As to how to perform cervicothoracic 3‐column osteotomy and instrumentation more safely and effectively, the advent of 3D printed models [23], O‐arm navigation [24], ultrasonic bone scalpel [25], and neuromonitoring [26] has enabled spinal surgeons to operate more safely, faster, and more effectively. With the assistance of these advanced techniques, only two patients in this study experienced neurological deficits, both of which significantly improved after 3 months of conservative treatment.

### 4.4 Strengths and Limitations

The strength of this study lies in the first introduction of the sequential correction technique for CTS patients and validating its short‐term effectiveness. Despite the promising outcome, our study has two limitations. First, due to the initial application of this sequential correction technique and the rarity of CTS, the sample size was relatively small. Second, the recruited patients in this study were very young with significant growth potential. A short‐term follow‐up is relatively insufficient to tell the real performance of this novel technique.

### 4.5 Prospects of Clinical Application

The sequential correction technique provides a more advantageous surgical treatment option for complex CTS patients, effectively overcoming the challenges of traditional two‐rod techniques during internal fixation implantation. In the future, this technique will be applied to more patients, with longer follow‐up periods to verify its long‐term efficacy.

## 5 Conclusion

This study offers a novel and superior choice in determining the instrumentation configuration of CTS‐HV during pre‐ and intra‐operative planning. The modified sequential correction technique enables simplified surgical maneuvers to be implemented following a step‐by‐step guide. It possesses the merits of easy rod installation, satisfying torticollis correction, good symmetry and verticality of the entire instrumentation, and high fixation rigidity with multi‐rod constructs across the cervicothoracic junction. Thus, it is endowed with great application value in the treatment of CTS.

## Author Contributions

Saihu Mao and Kai Sun: study design, data management and analysis, first draft and revision of the manuscript. Song Li and Jie Zhou: conceptualization, data management and analysis. Saihu Mao, Hongda Bao, Benlong Shi, Xu Sun, Zhen Liu, Yong Qiu and Zezhang Zhu: conceptualization, study design, surgical operation, manuscript revision. All authors have read and approved the final manuscript.

## Ethics Statement

This study was approved by the ethics committee of Nanjing Drum Tower Hospital (2021‐LCYJ‐DBZ‐05).

## Conflicts of Interest

The authors declare no conflicts of interest.
